# 865. Same Shift Susceptibility Testing of Cefepime-Taniborbactam and Cefiderocol Using The Selux Next-Generation Phenotyping System

**DOI:** 10.1093/ofid/ofad500.910

**Published:** 2023-11-27

**Authors:** David Jimenez, Kristin Baker, Benjamin Spears, Kelly Flentie, Kristen Roberts, Gabriel Michaels, Eric Stern

**Affiliations:** Selux Diagnostics, Charlestown, Massachusetts; Selux Diagnostics, Charlestown, Massachusetts; Selux Diagnostics, Charlestown, Massachusetts; Selux Diagnostics, Charlestown, Massachusetts; Selux Diagnostics, Charlestown, Massachusetts; Selux Diagnostics, Charlestown, Massachusetts; SeLux Diagnostics, Jamaica Plain, MA

## Abstract

**Background:**

Robust, efficient, and reliable antimicrobial susceptibility testing (AST) systems are an integral component of effective antibiotic treatment. Multi-drug resistant (MDR) bacteria continue to emerge at alarming rates, requiring continuous innovation and development of new antibiotics. Among the new promising treatments for severe bacterial infections caused by carbapenem-resistant gram-negative bacteria, Cefiderocol (FDC) and Cefepime-Taniborbactam (FTB) have emerged as two frontrunners. FDC is the only FDA-approved siderophore cephalosporin, and FTB pairs the fourth-generation cephalosporin Cefepime, with Taniborbactam, a novel beta-lactamase inhibitor. AST results are paramount when directing therapy for patients with MDR infections, yet there is an average 3-year delay between the approval of new antibiotics and 510k submissions for new agents on automated AST systems. This is typified by FDC, for which there are currently no FDA-cleared automated AST systems. Here we assess the accuracy of FDC and FTB AST results using the Selux Next-Generation Phenotyping System.

**Methods:**

For both FTB and FDC susceptibility, AST was performed using the Selux AST system and compared to CLSI broth microdilution (BMD) reference method in either cation-adjusted Muller Hinton broth (CAMHB) or its Iron depleted counterpart (ID-CAMHB). FDC, was tested in ID-CAMHB across 44 total CDC AR-Bank carbapenem-resistant K. pneumoniae, A. baumannii, and P. aeruginosa species. 34 CDC AR-Bank Strains predominantly resistant (30/34) to Cefepime were used to assess FTB. For FDC Essential Agreement (EA) and Categorical Agreement (CA) relative to ID-CAMHB were determined according to FDA breakpoints. FTB breakpoints were based on FDA breakpoints for Cefepime. For both agents, a comparison of BMD and the Selux system was accessed by EA and CA.

**Results:**

The Selux AST system produced MICs to FDC with an average accuracy of 98% EA and 98% CA in ID-CAMHB (Table 1). In FTB, the Selux AST system produced MICs with an average accuracy of 94% EA and 100% CA in CAMHB (Table 2).

**Figure:**
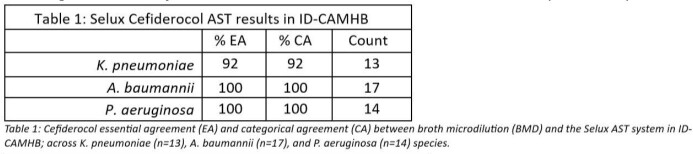

**Figure:**
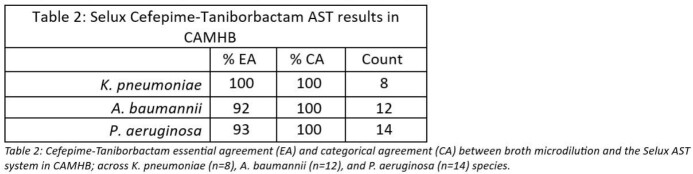

**Conclusion:**

These data demonstrate the ability of the Selux NGP system to provide accurate results for FDC and FTB. This is the first known demonstration of an automated AST system for these important new drugs.

**Disclosures:**

**David Jimenez, B.S.**, Selux Diagnostics: Employee **Kristin Baker, PhD**, Selux Diagnostics: Employee **Kelly Flentie, PhD**, Selux Diagnostics: Employee **Kristen Roberts, BS**, Selux Diagnostics: Stocks/Bonds **Gabriel Michaels, n/a**, Selux Diagnostics: Employee **Eric Stern, PhD**, Selux Diagnostics: Board Member|Selux Diagnostics: Ownership Interest

